# Use of Machine Learning Consensus Clustering to Identify Distinct Subtypes of Kidney Transplant Recipients With DGF and Associated Outcomes

**DOI:** 10.3389/ti.2022.10810

**Published:** 2022-12-08

**Authors:** Caroline C. Jadlowiec, Charat Thongprayoon, Napat Leeaphorn, Wisit Kaewput, Pattharawin Pattharanitima, Matthew Cooper, Wisit Cheungpasitporn

**Affiliations:** ^1^ Division of Transplant Surgery, Mayo Clinic, Phoenix, AZ, United States; ^2^ Division of Nephrology and Hypertension, Department of Medicine, Mayo Clinic, Rochester, MN, United States; ^3^ Renal Transplant Program, University of Missouri-Kansas City School of Medicine, Saint Luke’s Health System, Kansas City, MO, United States; ^4^ Department of Military and Community Medicine, Phramongkutklao College of Medicine, Bangkok, Thailand; ^5^ Department of Internal Medicine, Faculty of Medicine, Thammasat University, Pathum Thani, Thailand; ^6^ Medstar Georgetown Transplant Institute, Georgetown University, Washington, DC, United States

**Keywords:** kidney transplant, delayed graft function, clustering, machine learning, artificial intelligence

## Abstract

Data and transplant community opinion on delayed graft function (DGF), and its impact on outcomes, remains varied. An unsupervised machine learning consensus clustering approach was applied to categorize the clinical phenotypes of kidney transplant (KT) recipients with DGF using OPTN/UNOS data. DGF was observed in 20.9% (*n* = 17,073) of KT and most kidneys had a KDPI score <85%. Four distinct clusters were identified. Cluster 1 recipients were young, high PRA re-transplants. Cluster 2 recipients were older diabetics and more likely to receive higher KDPI kidneys. Cluster 3 recipients were young, black, and non-diabetic; they received lower KDPI kidneys. Cluster 4 recipients were middle-aged, had diabetes or hypertension and received well-matched standard KDPI kidneys. By cluster, one-year patient survival was 95.7%, 92.5%, 97.2% and 94.3% (*p* < 0.001); one-year graft survival was 89.7%, 87.1%, 91.6%, and 88.7% (*p* < 0.001). There were no differences between clusters after accounting for death-censored graft loss (*p* = 0.08). Clinically meaningful differences in recipient characteristics were noted between clusters, however, after accounting for death and return to dialysis, there were no differences in death-censored graft loss. Greater emphasis on recipient comorbidities as contributors to DGF and outcomes may help improve utilization of DGF at-risk kidneys.

## Graphical Abstract

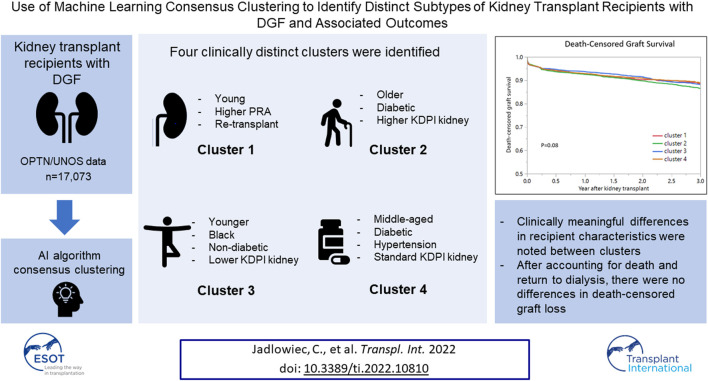

## Introduction

Delayed graft function (DGF) is common following kidney transplantation (KT) and its incidence varies anywhere from ≤30% in standard kidney donor profile index (KDPI) kidneys to upwards of 60% in kidneys allografts coming from donation after circulatory death (DCD), severe acute kidney injury (AKI), and high KDPI (KDPI ≥85%) donor (1–4). Although the definition of DGF, need for dialysis within 7 days of KT, is simplistic and allows for consistency, the reporting of DGF as a binary outcome in data analyses fails to capture complex clinical nuances that contribute to outcomes. Donor-related characteristics, such as DCD status and acute kidney injury, are commonly identified risk-factors for DGF, although recipient-specific characteristics and transplant events also play significant roles and influence outcomes (1–3, 5, 6). Published data and transplant community opinion on DGF, and its impact on outcomes, remains varied. Many studies have shown an association between DGF and inferior survival (7–9). While other studies have shown that select DGF subgroups have equivocal outcomes compared to those with primary function (1–4). The observed inconsistencies in DGF outcomes are possibly related to how DGF data is analyzed, with many studies focusing on predetermined individual donor-, recipient-, or transplant characteristics rather than a balanced interpretation of competing variables (1–9).

Artificial intelligence and machine learning (ML) function as clinical decision support tools have been used to help individualize patient care, including organ transplantation (10–15). Unsupervised consensus clustering, a type of ML, can be applied to clinical data and its application has allowed for the discovery of novel data patterns and distinct subtypes (16–18). It has facilitated the discovery of similarities and heterogeneities among data variables and has also distinguished data into clinically meaningful clusters independent of predefined risk-variables (16, 17). Recent studies have demonstrated that distinct subtypes identified by ML consensus clustering approach can forecast different clinical outcomes (19–21). To better understand differing DGF outcomes, we used an unsupervised ML consensus clustering approach to categorize clinical phenotypes of KT recipients with DGF and their paired donors.

## Materials and Methods

Adult patients who received a kidney-only transplant in the United States from 2015 to 2019 were identified using the Organ Procurement and Transplantation Network (OPTN)/United Network for Organ Sharing (UNOS) database. All KT patients with DGF were included. DGF was defined as the need for dialysis within 7 days after KT. Multivisceral transplant recipients were not included in this dataset. After accounting for all adult kidney-only transplant recipients (*n* = 81,548), adult kidney-only transplant recipients without DGF (*n* = 64,475) were excluded. The Mayo Clinic Institutional Review Board approved this study (IRB 21-007698).

Recipient-, donor-, and transplant-related variables shown in Table 1, in addition to recipient ABO, positive hepatitis C serostatus, hepatitis B surface antigen, human immunodeficiency virus serostatus, working income, public insurance, United States resident, undergraduate education or higher, serum albumin, ABO incompatibility, Ebstein-Barr and cytomegalovirus status, were abstracted from the OPTN/UNOS database. All variables had ≤5% missing data (Supplementary Table S1). We imputed missing data using multiple imputation by chained equation (MICE) method (12). One-year acute rejection was defined as clinical acute rejection, independent of chronic rejection, occurring within the first-year post-transplantation as reported to UNOS.

**TABLE 1:** Clinical characteristics, according to clusters, of kidney transplant recipients with DGF.

	All (*n* = 17,073)	Cluster 1 (*n* = 1,891)	Cluster 2 (*n* = 6,918)	Cluster 3 (*n* = 5,442)	Cluster 4 (*n* = 2,822)	*p*-value
Recipient Characteristics						
Age (year)	54.1 ± 12.6 (56)	47.2 ± 12.6 (48)	61.5 ± 8.3 (62)	45.9 ± 11.6 (46)	56.3 ± 11.5 (58)	<0.001
Male sex	11475 (67%)	1199 (63%)	4854 (70%)	3746 (69%)	1676 (59)	<0.001
Race						<0.001
White	5208 (30%)	753 (40%)	2022 (29%)	1167 (21%)	1266 (45%)	
Black	6645 (39%)	681 (36%)	2627 (38%)	2692 (49%)	645 (23%)	
Hispanic	3506 (21%)	324 (17%)	1464 (21%)	1059 (20%)	659 (23%)	
Other	1714 (10%)	133 (7%)	805 (12%)	524 (10%)	252 (9%)	
Body mass index (kg/m^2^)	29.3 ± 5.5 (29.0)	27.5 ± 5.6 (27.0)	30.1 ± 5.2 (29.9)	28.8 ± 5.8 (28.2)	29.7 ± 5.3 (29.4)	<0.001
No. of kidney transplant(s)	1.1 ± 0.4	2.1 ± 0.4	1.0 ± 0.1	1.0 ± 0.1	1.0 ± 0.1	<0.001
PRA, median (IQR)	0 (0, 39)	98 (83, 100)	0 (0, 3)	0 (0, 16)	0 (0, 57)	<0.001
Dialysis duration						<0.001
Preemptive	610 (4%)	74 (4%)	225 (3%)	183 (3%)	128 (5%)	
<1 year	1054 (6%)	126 (7%)	406 (6%)	302 (6%)	220 (8%)	
1–3 years	3120 (18%)	445 (23%)	1199 (17%)	734 (13%)	742 (26%)	
>3 years	12289 (72%)	1246 (66%)	5088 (74%)	4223 (78%)	1732 (61%)	
Cause of kidney disease						<0.001
Diabetes mellitus	5998 (35%)	74 (4%)	4163 (60%)	600 (11%)	1161 (41%)	
Hypertension	4151 (24%)	171 (9%)	1300 (19%)	2101 (39%)	579 (21%)	
Glomerular disease	2780 (16%)	313 (16%)	595 (9%)	1443 (27%)	429 (15%)	
PKD	976 (6%)	35 (2%)	302 (4%)	406 (7%)	233 (8%)	
Other	3168 (19%)	1298 (69%)	558 (8%)	892 (16%)	420 (15%)	
Comorbidities						
Diabetes mellitus	7404 (43%)	349 (18%)	4788 (69%)	901 (17%)	1366 (48%)	<0.001
Malignancy	1584 (9%)	213 (11%)	766 (11%)	316 (6%)	289 (10%)	<0.001
PVD	1941 (11%)	144 (8%)	1159 (17%)	304 (6%)	334 (12%)	<0.001
Functional status						<0.001
10–30%	53 (0%)	2 (0%)	30 (1%)	13 (0%)	8 (0%)	
40–70%	8789 (52%)	872 (46%)	3829 (55%)	2609 (48%)	1479 (53%)	
80–100%	8231 (48%)	1017 (54%)	3059 (44%)	2820 (52%)	1335 (47%)	
Donor Characteristics						
Kidney donor status						<0.001
Non-ECD	13528 (79%)	1697 (90%)	4530 (65%)	5161 (95%)	2140 (76%)	
ECD	2778 (16%)	145 (8%)	2160 (31%)	61 (1%)	412 (15%)	
Living donor	767 (5%)	49 (3%)	228 (3%)	220 (4%)	270 (10%)	
Age	41.4 ± 14.6 (43)	37.9 ± 13.5 (39)	49.5 ± 11.0 (51)	31.3 ± 13.1 (31)	43.2 ± 12.9 (45)	<0.001
Male sex	10571 (62%)	1223 (65%)	4057 (59%)	3565 (65%)	1726 (61%)	<0.001
Race						<0.001
White	11691 (68%)	1258 (66%)	4804 (69%)	3575 (66%)	2054 (73%)	
Black	2247 (13%)	258 (14%)	924 (13%)	836 (15%)	229 (8%)	
Hispanic	2350 (14%)	290 (15%)	841 (12%)	810 (15%)	409 (14%)	
Other	785 (5%)	85 (4%)	349 (5%)	221 (4%)	130 (5%)	
Hypertension	5678 (33%)	516 (27%)	3401 (49%)	829 (15%)	932 (33%)	<0.001
KDPI						<0.001
Living donor	767 (4%)	49 (3%)	228 (3%)	220 (4%)	270 (9%)	
KDPI<85	14611 (86%)	1795 (95%)	5265 (76%)	5160 (95%)	2391 (85%)	
KDPI≥85	1695 (10%)	47 (2%)	1425 (21%)	62 (1%)	161 (6%)	
Transplant-Related Characteristics						
HLA mismatch ABDR	4 (4, 5)	3 (2, 4)	5 (4, 5)	5 (4, 5)	3 (2, 3)	<0.001
CIT (hours)	19.0 ± 9.3 (18.4)	19.6 ± 8.5 (19.3)	20.3 ± 9.6 (19.4)	17.3 ± 8.8 (16.4)	18.5 ± 9.8 (18.6)	<0.001
Kidney on pump	8280 (48%)	701 (37%)	3961 (57%)	2396 (44%)	1222 (43%)	<0.001
Allocation type						<0.001
Local	10996 (64%)	752 (40%)	4347 (63%)	4208 (77%)	1689 (60%)	
Regional	2748 (16%)	325 (17%)	1437 (21%)	574 (11%)	412 (15%)	
National	3329 (20%)	814 (43%)	1134 (16%)	660 (12%)	721 (25%)	
Induction Immunosuppression						
Thymoglobulin	10777 (63%)	1425 (75%)	4136 (60%)	3478 (64%)	1738 (62%)	<0.001
Alemtuzumab	2651 (15%)	270 (14%)	973 (14%)	965 (18%)	443 (16%)	<0.001
Basiliximab	3308 (19%)	122 (6%)	1744 (25%)	877 (16%)	565 (20%)	<0.001
Other	240 (1%)	27 (1%)	105 (1%)	65 (1%)	43 (1%)	0.44
No induction	965 (6%)	87 (5%)	404 (6%)	310 (6%)	164 (6%)	0.21
Maintenance Immunosuppression						
Tacrolimus	15513 (91%)	1742 (92%)	6250 (90%)	4958 (91%)	2563 (91%)	0.10
Cyclosporine	152 (1%)	25 (1%)	56 (1%)	47 (1%)	24 (1%)	0.20
Mycophenolate	15678 (92%)	1746 (92%)	6329 (92%)	5020 (92%)	2583 (92%)	0.35
Azathioprine	61 (0%)	10 (1%)	21 (0%)	19 (0%)	11 (0%)	0.53
mTOR inhibitors	46 (0%)	4 (0%)	24 (0%)	9 (0%)	9 (0%)	0.24
Steroid	12337 (72%)	1523 (81%)	4875 (70%)	3930 (72%)	2009 (71%)	<0.001

### Clustering Analysis

An unsupervised ML was applied by conducting a consensus clustering approach to categorize clinical phenotypes of KT recipients with DGF (13). A pre-specified subsampling parameter of 80% with 100 iterations and the number of potential clusters (k) ranging from 2 to 10 were used to avoid producing an excessive number of clusters that would not be clinically useful. The optimal number of clusters was determined by examining the consensus matrix (CM) heat map, cumulative distribution function (CDF), cluster-consensus plots with the within-cluster consensus scores, and the proportion of ambiguously clustered pairs (PAC). The within-cluster consensus score, ranging between 0 and 1, was defined as the average consensus value for all pairs of individuals belonging to the same cluster (14). A value closer to one indicates better cluster stability. PAC, ranging between 0 and 1, was calculated as the proportion of all sample pairs with consensus values falling within the predetermined boundaries (15). A value closer to zero indicates better cluster stability (16). To avoid cherry picking results, we used validated clustering approaches including examination of the consensus matrix (CM) heat map, cumulative distribution function (CDF), cluster-consensus plots with the within-cluster consensus scores, and the proportion of ambiguously clustered pairs (19, 21–23). The detailed consensus cluster algorithms used in this study for reproducibility are provided in Online Supplementary.

### Outcomes

Outcomes identified included acute rejection within the first post-transplant year and 1- and 3-year patient, kidney allograft and death-censored graft survival.

### Statistical Analysis

After each KT recipient with DGF was assigned a cluster using the consensus clustering approach, we performed a comparison of clinical characteristics and posttransplant outcomes among the assigned clusters. Clinical characteristics among the assigned clusters were compared using Chi-squared analysis for categorical variables and analysis of variance (ANOVA) for continuous variables. The key characteristics of each cluster were identified using the standardized mean difference between each cluster and the overall cohort with the pre-specified cut-off of >0.3. The cumulative risks of death-censored graft failure and death after KT were estimated using Kaplan-Meier analysis, and the risks among the assigned cluster were compared using Cox proportional hazard analysis. As OPTN/UNOS only reported whether allograft rejection occurred within 1 year after KT but did not specify the occurrence date, we compared the risk of 1-year acute allograft rejection among the assigned clusters using logistic regression analysis. We did not adjust the association of the assigned cluster and posttransplant outcomes in multivariable analysis for difference in baseline characteristics because unsupervised consensus clustering approach purposefully generated clinically distinct clusters. R, version 4.0.3 (RStudio, Inc., Boston, MA; http://www.rstudio.com/) was used for statistical analyses; ConsensusClusterPlus package (version 1.46.0) for consensus clustering analysis, and the MICE command in R for multivariable imputation by chained equation (24).

## Results

During this study period, a total of 81,548 adult patients received a KT, and of those, 20.9% (*n* = 17,073) had DGF. Consensus clustering analysis was performed on the 17,073 KT recipients with DGF.

**FIGURE 1:**
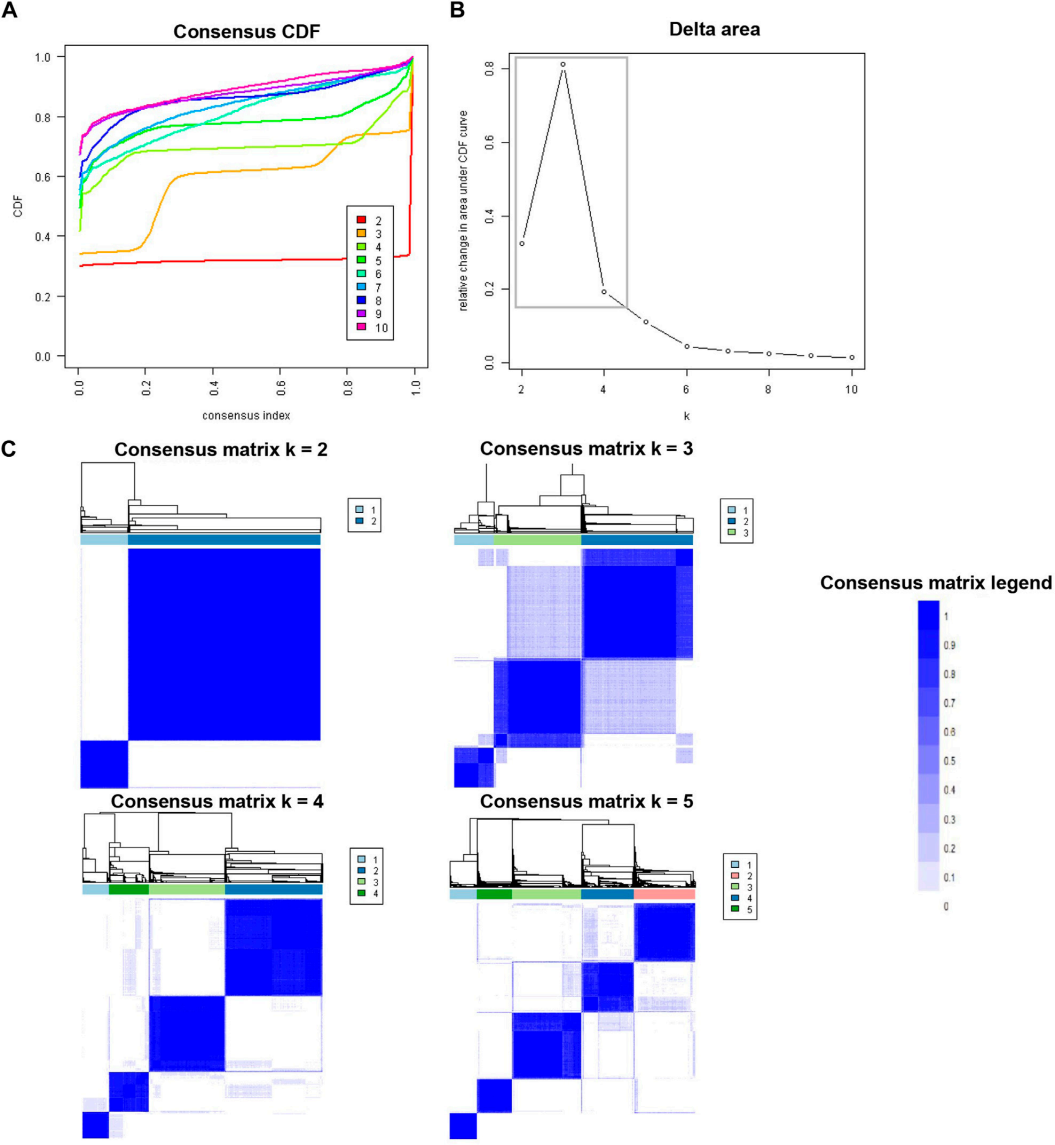
**(A)** CDF plot displaying consensus distributions for each k; **(B)** Delta area plot reflecting the relative changes in the area under the CDF curve. **(C)** Consensus matrix heat map depicting consensus values on a white to blue color scale of each cluster.

**FIGURE 2:**
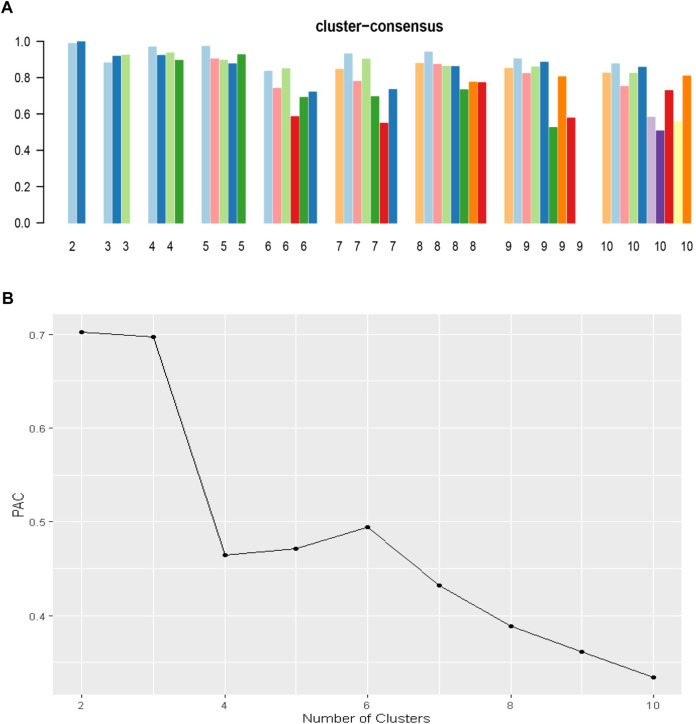
**(A)** The bar plot represents the mean consensus score for different numbers of clusters (K ranges from two to ten); **(B)** The PAC values assess ambiguously clustered pairs.

Figure 1A shows the CDF plot consensus distributions for each cluster of KT recipients with DGF; the delta area plot shows the relative change in the area under the CDF curve (Figure 1B). The largest changes in area occurred between k = 2 and k = 4, at which point the relative increase in area became noticeably smaller. As shown in the CM heat map (Figure 1C), the ML algorithm identified cluster 2 and cluster 4 with clear boundaries, indicating good cluster stability over repeated iterations. The mean cluster consensus score was comparable between k = 2 and k = 4 (*p* > 0.05) (Figure 2A). Favorable low PAC was demonstrated for 4 clusters than 2 clusters (Figure 2B). Thus, using baseline variables at the time of transplant, the consensus clustering analysis identified 4 clusters that best represented the data pattern of our KT recipients with DGF.

### Clinical Characteristics of DGF Clusters

Table 1 shows recipient-, donor-, and transplant-related characteristics of included patients. DGF was observed in 20.9% of kidney transplants (*n* = 17,073) that occurred during this study period. The majority of recipients with DGF were male (67%, *n* = 11,475) and had more than 3 years of time on dialysis (72%, *n* = 12,289). Most kidneys with DGF were non-extended criterion donor (ECD) (79%, *n* = 13,528) standard KDPI kidneys (86%, *n* = 14,611). Donors of kidneys with DGF had a median age of 43 years, were likely to be male (62%, *n* = 10,571), white (68%, *n* = 11,691), be transplanted by local centers (64%, *n* = 10,996), and have a median CIT of 18.4 h.

**FIGURE 3:**
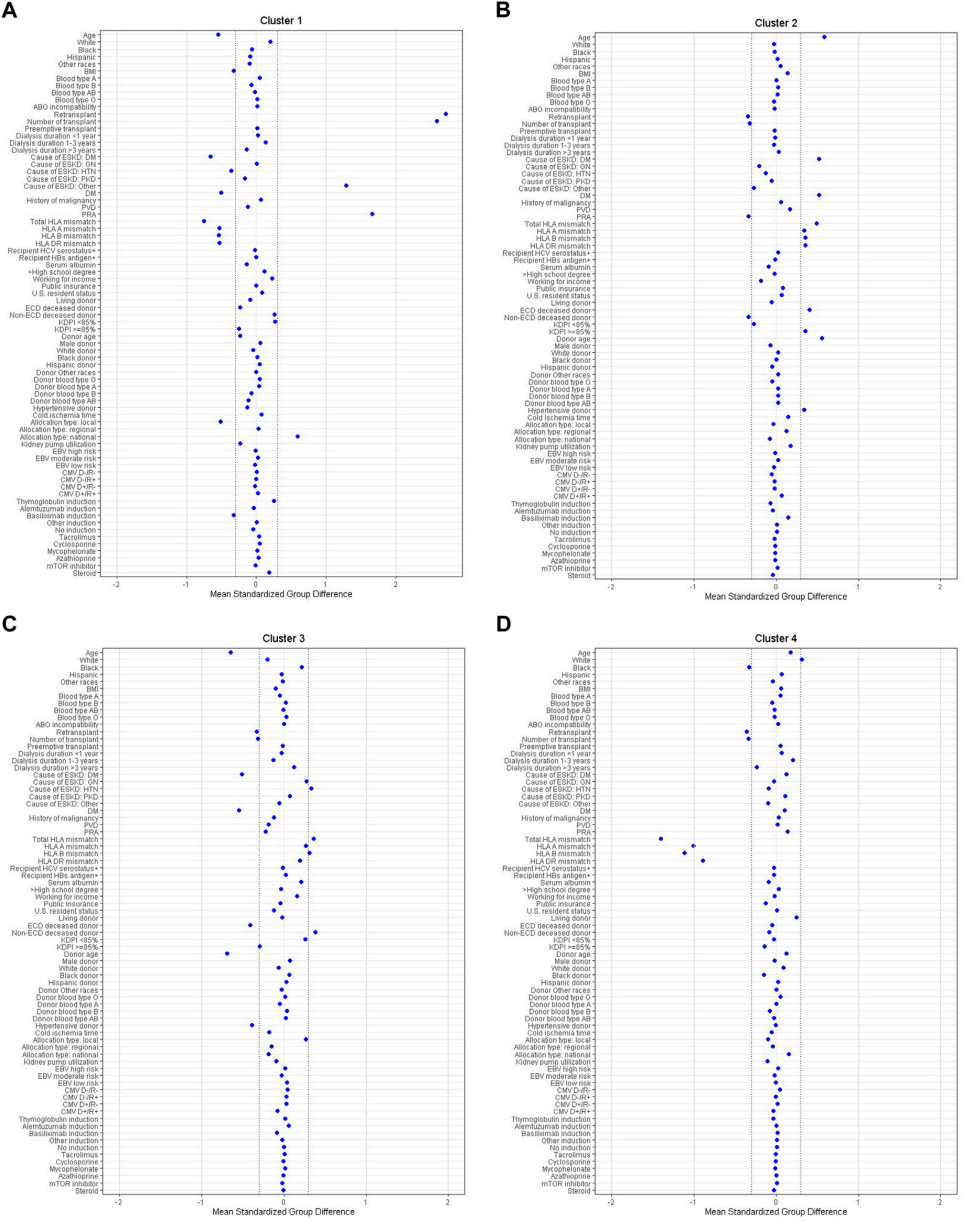
**(A–D)** The standardized differences in Clusters 1–4 of DGF for each of baseline parameters. The x axis is the standardized differences value, and the y axis shows baseline parameters. The dashed vertical lines represent the standardized differences cutoffs of <−0.3 or >0.3. Abbreviations: BMI, body mass index; CMV, cytomegalovirus; D, donor; DGF, delayed graft function; DM, diabetes mellitus; EBV, Epstein-Barr virus; ECD, extended criteria donor; ESKD, end stage kidney disease; GN, glomerulonephritis; HBs, hepatitis B surface; HCV, hepatitis C virus; HIV, human immunodeficiency virus; HLA, human leucocyte antigen; HTN, hypertension; KDPI, kidney donor profile index; mTOR, mammalian target of rapamycin; PKD, polycystic kidney disease; PRA, panel reactive antibody; PVD, peripheral vascular disease; R, recipient.

Within this group of 17,073 recipients with DGF, consensus clustering analysis identified four distinct clinical clusters as shown in Table 1. There were 1,891 (11%) patients in cluster 1, 6,918 (41%) patients in cluster 2, 5,442 (32%) patients in cluster 3, and 2,822 (17%) patients in cluster 4. According to standardized mean differences, shown in Figure 3, cluster 1 was characterized by younger (median age 48 years), low BMI, non-diabetic, kidney re-transplant recipients who had a high PRA, a low number of HLA mismatches, and received depleting induction. Cluster 1 recipients received standard KDPI kidneys (95% had a KDPI score <85%, *n* = 1795) and had the highest percentage of nationally allocated kidneys (43%, *n* = 814).

By comparison, cluster 2 recipients were the oldest (median age 62 years) of the four clusters. They had a higher BMI (30.1 ± 5.2 kg/m^2^) and were likely to be diabetic (69%, *n* = 4,788) with the majority (74%, *n* = 5,088) having ≥3 years of dialysis time. Cluster 2 recipients were not sensitized. They were first-time KT recipients with a high number of HLA mismatches. Cluster 2 had more recipients with lower functional status, with 55% having a Karnofsky score between 40-70%. Out of the four clusters, cluster 2 recipients were the most likely to receive an ECD (31%, *n* = 2,160), high KDPI (21%, *n* = 1,425) kidney, although the majority (76%, *n* = 5,265) received standard KDPI kidneys. Peripheral vascular disease (PVD) was present in 17% of cluster 2 recipients.

Cluster 3 recipients were young in age (median age 46 years) and non-diabetic. They were more likely to be black (49%, *n* = 2,696) and have hypertension (39%, *n* = 2,101). Similar to cluster 2, they were also first-time KT recipients with a high number of HLA mismatches and a low PRA. They were unlikely to receive an ECD (1%, *n* = 61), high KDPI (1%, *n* = 62) kidney. Instead, the majority of cluster 3 recipients received standard KDPI kidneys (76%, *n* = 5,265), from young (median age 31 years), non-hypertensive donors. These kidneys came from local donors (77%, *n* = 4,208). Cluster 3 kidneys had the shortest CIT (median 16.4 h).

Lastly, cluster 4 recipients were middle aged (median age 58 years), first-time KT recipients with greater than 3 years of dialysis times, a low PRA, and a lower number of HLA mismatches. Recipients in cluster 4 were likely to have kidney disease as a result of diabetes (41%) or hypertension (21%). Forty-eight percent (*n* = 1,366) were diabetic and 12% (*n* = 334) had PVD. Recipient functional status was also lower in cluster 4, with 53% of recipients having a Karnofsky score between 40%–70%. The majority received non-ECD (76%, *n* = 2,140), standard KDPI (85%, *n* = 2,391) kidneys that largely came from local donors (60%, *n* = 1,889).

### Posttransplant Outcomes of DGF Clusters

**FIGURE 4:**
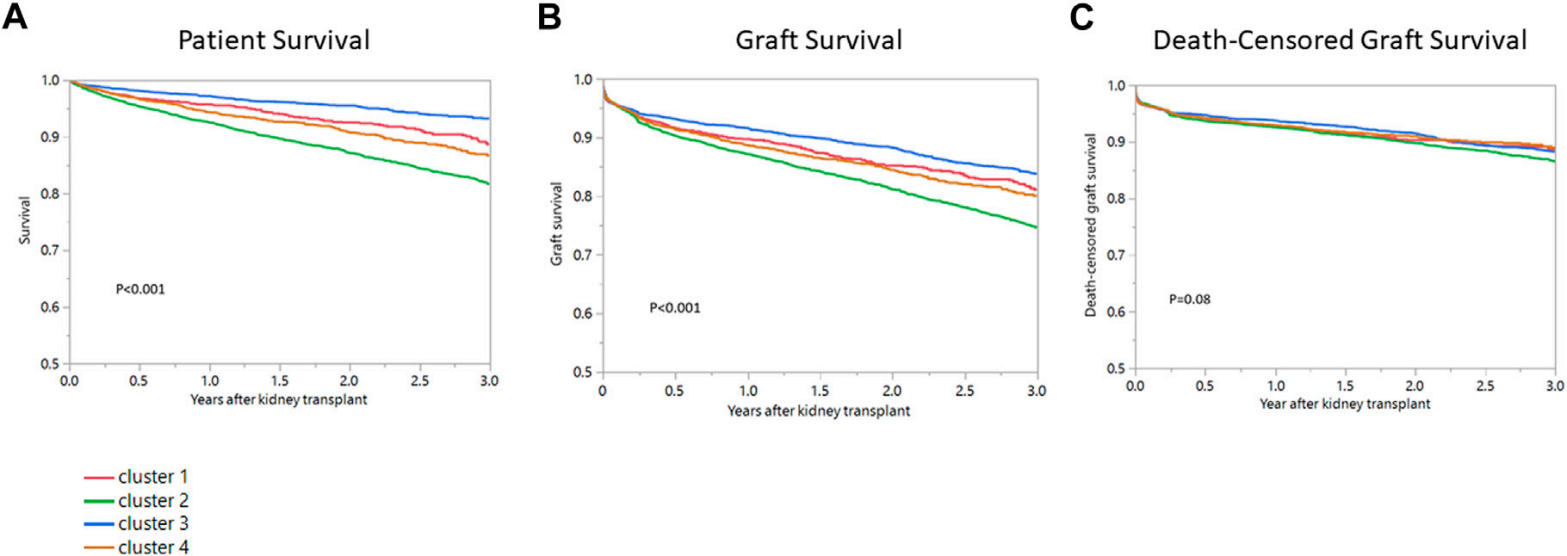
**(A)** Patient survival, **(B)** Graft survival, **(C)** Death-censored graft survival after kidney transplant among four clusters of kidney transplant recipients with DGF in the United States.

Table 2 and Figure 4 show cluster-based posttransplant outcomes. Median follow-up time for patient survival was 412 days (IQR 199-971). Median follow-up time for graft survival was 391 days (IQR 188-945). One-year patient survival in clusters 1, 2, 3 and 4 was 95.7%, 92.5%, 97.2% and 94.3%. Cluster 3 had the most favorable patient survival (ref) with cluster 2 (HR 2.66, 95% CI 2.19–3.24) having the worst (*p* < 0.001) (Table 2, Figure 4A). One-year graft survival in clusters 1, 2, 3 and 4 was 89.7%, 87.1%, 91.6%, 88.7% (Table 2, Figure 4B). Similar to patient survival, cluster 3 recipients had the best 1-year graft survival (ref) with cluster 2 (HR 1.54, 95% 1.35-1.71) recipients having the worst (*p* < 0.001). One-year death-censored graft survival in clusters 1, 2, 3 and 4 was 92.7%, 92.5%, 93.7%, and 92.9% (Table 2, Figure 4C) and there were no differences in death-censored graft survival when comparing clusters (*p* < 0.08).

**TABLE 2:** Posttransplant outcomes, according to clusters, of kidney transplant recipients with DGF.

	Cluster 1	Cluster 2	Cluster 3	Cluster 4
1-Year				
Patient survival	95.7% (1.55, 1.17–2.07)	92.5% (2.66, 2.19–3.24)	97.2% (1, ref)	94.3% (1.98, 1.56–2.52)
Graft survival	89.7% (1.22, 1.03–1.46)	87.1% (1.52, 1.35–1.71)	91.6% (1, ref)	88.7% (1.33, 1.15–1.55)
Death-censored graft survival	92.7% (1.15, 0.94–1.41)	92.5% (1.18, 1.03, 1.36)	93.7% (1, ref)	92.9% (1.12, 0.93–1.34)
1-year acute rejection	10.2% (2.86, 2.24–3.64)	5.3% (1.42, 1.14–1.76)	7.0% (1.90, 1.52–2.36)	3.8% (1, ref)
3-Year				
Patient survival	88.7% (1.63, 1.32–2.03)	81.6% (2.78, 2.39–3.24)	93.2% (1, ref)	86.7% (1.98, 1.64–2.39)
Graft survival	81.1% (1.20, 1.04–1.39)	74.6% (1.58, 1.43–1.75)	83.8% (1, ref)	80.0% (1.29, 1.14–1.47)
Death-censored graft survival	88.6% (1.05, 0.88–1.26)	86.5% (1.16, 1.03–1.32)	88.2% (1, ref)	88.9% (1.03, 0.87–1.20)

One-year acute rejection in clusters 1, 2, 3 and 4 was 10.2%, 5.3%, 7.0%, 3.0% (Table 2). Cluster 4 had the lowest observed acute rejection within the first-year post-transplant (ref). Clusters 1 (HR 2.86, 2.24–3.64) and 3 (HR 1.90, 95% CI 1.52–2.36) had the highest number of reported acute rejection events.

## Discussion

The clinical significance of DGF and its impact on KT outcomes continues to be debated and some of the reported variation in outcomes is likely a reflection of how DGF data is analyzed (1–9). The interpretation of DGF data remains heavily influenced as a result of predefined study constructs based on fixed and isolated donor-, recipient-, and transplant characteristics, such as donor DCD status, CIT, or rejection (1–9). To better understand differing DGF outcomes and viewpoints, we used an unsupervised ML consensus clustering approach to categorize the clinical phenotypes of KT recipients with DGF and their paired donors.

During this recent study period, the overall incidence of DGF in the US was 20.9%. The majority of recipients with DGF were males who were on dialysis ≥3 years and who received non-ECD, standard KDPI kidneys. Within this group of 17,073 recipients with DGF, consensus clustering analysis identified four distinct clinical clusters. Cluster 1 was characterized by younger, low BMI, non-diabetic, kidney re-transplant recipients who had a high PRA. Cluster 2 recipients were the oldest of the four clusters, had a higher BMI, were likely to have lower functional status, and be diabetic with 3+ years of dialysis vintage. They were also the most likely to receive ECD high KDPI kidneys. Cluster 3 recipients were young and non-diabetic. They were more likely to be black, have hypertension and receive higher HLA mismatched, lower KDPI kidneys. Lastly, cluster 4 recipients were middle-aged, first-time KT recipients with either diabetes or hypertension, lower functional status, dialysis duration ≥3 years, and a low PRA. Patient and graft survival varied by cluster, however, after accounting for death with a functioning graft, there were nosurvival differences between the four clusters suggesting that recipient comorbidities played an important role in graft outcomes (Figure 4C).

Although DGF is often attributed to donor quality and CIT, the majority of kidney allografts used during this study period came from non-ECD, standard KDPI, younger donors with a median CIT of 18.4 h (1–3, 5, 6). Only a small percentage of donors had hypertension, and the majority of kidneys were transplanted locally. Clinically significant differences in recipient comorbidities were notable between the clusters. Cluster 1 recipients were highly sensitized re-transplants, cluster 2 recipients were older diabetics, cluster 3 recipients were young non-diabetic black first-time transplants with hypertension, and cluster 4 recipients were predominantly middle-aged, recipients with diabetes or hypertension and lower functional status. As might be predicted, patient survival was best in 3 and lower in clusters 2 and 4. Despite varying cluster-specific recipient comorbidities, there were however no difference in death-censored graft survival between the four clusters.

The lack of difference in death-censored graft loss suggests that different factors contributed to survival across the four clusters. Recipient comorbidities, such as diabetes, dialysis vintage, PVD and dialysis vintage, likely played a significant role for clusters 2 and 3. Lack of difference in death-censored graft loss between clusters 2 and 4 suggests that there is increased room to increase use of ECD and high KDPI allografts for patients with these demographics. High KDPI kidneys continue to be at significant risk of discard and recipients with demographics shown in cluster 2 and 4 are well suited for these allografts (24). Although recipients in cluster 4 received more standard KDPI low HLA mismatched allografts, ultimately there were no differences in death-censored graft survival. Although cluster 1 recipients were younger in age and had less comorbidities, they were sensitized re-transplants. They carried the highest risk for rejection and likely had decreased survival as a result of risk factors such as infection due to over-immunosuppression, rejection as a result of infection or reactivation of preexisting donor specific antibodies or recurrent disease. Outcomes related to cluster 3 recipients were possibly the most surprising. Based on comorbidities, these recipients would perhaps be predicted to have the best outcomes. This finding possibly underscores that racial disparities in transplant impact outcomes and that variables, such as risk for rejection, socioeconomic barriers and access to healthcare, disproportionately affect minorities (21, 25). Although graft quality, demonstrated though use of predominantly standard KDPI allografts was observed in cluster 4, better HLA matching, need for a more personalized approach to immunosuppression or better post-transplant support, might result in improved outcomes.

DGF is often felt to be a risk factor for early acute rejection (7, 26). The overall incidence of acute rejection post-transplant has been reported to range between 10% and 29% with the inclusion of subclinical rejection (27, 28). In this study, the reported incidence of acute rejection was low ranging from 3.8% to 10.2% with the majority of recipients, regardless of PRA or age, received depleting induction. Although historically rejection data as reported in UNOS has had limitations due to underreporting, the use of depleting induction remains a widespread practice preference in the United States and these lower rejection rates may be reflective of several factors (21, 30). Increasingly, many centers are moving towards earlier initiation of CNIs in combination with use of depleting therapy in the setting of DGF to minimize this early rejection occurrences (1–4). The highest incidence of acute rejection was observed in cluster 1 recipients who were highly sensitized re-transplants. Despite this being an *at-risk* group for rejection, the reported incidence was only 10.2%. Cluster 3 recipients had the second highest reported incidence of acute rejection at 7.0%. Although this group was not sensitized, risk factors such as young recipient age, black race, and high HLA mismatches may have played a role in the higher number of rejection events (3, 30–33). Cluster 2 recipients were the oldest and the most likely to receive ECD high KDPI kidneys and also receive non-depleting induction. While cluster 2 was possibly at higher risk for a longer duration of DGF due to recipient and donor characteristics, there was not an increase in acute rejection episodes noted. The results from this analysis suggest that the overall incidence of acute rejection for kidneys with DGF is low (25–29).

In using the OPTN/UNOS national registry data, there are several limitations. This clustering analysis included only recipients with DGF. As such, there is not a comparison group for similarly matched recipients and donors without DGF. Because of the registry nature of this study, there is lack of detail regarding exact causes for DGF, mortality and graft loss. We also do not know the outcomes for mate kidneys from the same donor. Missing data remains an inherent limitation of the UNOS dataset. Although we acknowledge this as a limitation, all variables in our study had missing data <5%, and it is unlikely that missing data imputation substantially altered the results of our analysis. Additionally, we acknowledge that the current working definition of DGF has inherent limitations such that it is simplistic and does not account for additional complexities, such as DGF duration and oliguria. Forthcoming updated guidelines in terminology specific to DGF will be helpful in addressing these current limitations. These limitations highlight the need for better reporting practices specific to DGF. Lastly, although unsupervised ML clustering applied in this study provided detailed information on distinct phenotypes and outcomes pertaining to kidney transplant recipients with DGF, the clinical characteristics attributed to the clusters were not necessarily novel and unsupervised ML clustering approaches have limitations in that they do not directly generate risk prediction for each individual. Future studies using supervised ML prediction models to predict outcomes of kidney transplant recipients with DGF are needed for validation.

Despite these limitations, the interpretation of DGF data to date remains heavily influenced as a result of predefined study constructs. Unsupervised clustering machine learning algorithms help us understand the characteristics of different clusters of kidney transplant patients with DGF within the current transplant practice in the U.S., and the algorithms do not use labeled outcomes. Unlike supervised machine learning models, unsupervised machine learning models do not have issues with overfitting and do not have limitations of variables in the clustering algorithms. To our knowledge however, this is the first ML clustering approach to look at the impact of DGF on KT outcomes. Outcomes specific to DGF have been varied and have often been reported as isolated analyses focusing on individual donor-, recipient-, or transplant characteristics rather than isolated interpretation of competing variables. By applying a ML clustering approach, this study has allowed for an unbiased assessment of KT outcomes for those with DGF.

Clinical outcomes specific to DGF are currently described in a binary fashion, however factors contributing to DGF are complex, nonbinary and varied. Significant variation exists between different studies reporting on DGF and much of this variation can be accounted for by differences in analyses. In this study, unsupervised ML was applied to KT recipients with DGF and their paired donors and this resulted in the identification of four clinically distinct clusters with differing post-transplant outcomes. The majority of kidneys utilized in the United States continue to come from standard KDPI non-ECD donors and more obvious clinical heterogeneity is notable in cluster-specific recipient comorbidities. The majority of kidneys with DGF in the United States come from standard KDPI donors. Clinically meaningful differences in recipient characteristics were noted between clusters, and, after accounting for death and return to dialysis, there were no differences in death-censored graft loss. Immunologic, cardiac, metabolic, and socioeconomic contributors likely play significant roles in varying outcomes and, although DGF is a predefined clinical endpoint, recipient comorbidities assume an important role in survival outcomes.

## Data Availability

The datasets presented in this study can be found in online repositories. The names of the repository/repositories and accession number(s) can be found below: The Organ Procurement and Transplantation Network (OPTN)/United Network for Organ Sharing (UNOS) database (accession number: DATA0006605).

## Abbreviations

ANOVA, analysis of variance; BMI, body mass index; CIT, cold ischemia time; DGF, delayed graft function; ECD, extended criterion donor; HLA, human leukocyte antigen; HTN, hypertension; KDPI, kidney donor profile index; KT, kidney transplant; MICE, multiple imputation by chained equation; ML, machine learning; mTOR, mammalian target of rapamycin; OPTN, Organ Procurement and Transplantation Network; PRA, panel reactive antibody; PVD, peripheral vascular disease; UNOS, United Network for Organ Sharing.
