# Supplementary material for: Use of Machine Learning Consensus Clustering to Identify Distinct Subtypes of Kidney Transplant Recipients With DGF and Associated Outcomes
Source: Transpl Int. 2022 Dec 8;35:10810. doi: 10.3389/ti.2022.10810 (PMC9773391; doi:10.3389/ti.2022.10810)
Supplement: Supplementary file 1 [file Table1.pdf]

Table S1 – Missing Data

	Missing data (total=17,073)
Recipient Age	0 (0)
Recipient sex	0 (0)
Recipient race	0 (0)
ABO blood group	0 (0)
Body mass index	0 (0)
Kidney retransplant	0 (0)
Kidney donor status	0 (0)
Dialysis duration	70 (0.4)
Cause of end-stage kidney disease	0 (0)
Comorbidity	
- Diabetes mellitus	7 (0.04)
- Malignancy	0 (0)
- Peripheral vascular disease	142 (0.8)
PRA (%)	489 (2.9)
HCV serostatus	0 (0)
HBs antigen	0 (0)
HIV serostatus	0 (0)
Functional status	491 (2.9)
Working income	820 (4.8)
Insurance	1 (0.01)
US resident	1 (0.01)
Education	393 (2.3)
Serum albumin	706 (4.1)
Donor age	0 (0)
Donor sex	0 (0)
Donor race	0 (0)
History of hypertension in donor	0 (0)
KDPI	1 (0.01)
HLA mismatch	17 (0.1)
Cold ischemia time	75 (0.4)
Kidney on pump	0 (0)
Allocation type	0 (0)
EBV status	679 (4.0)
CMV status	0 (0)
Induction immunosuppression	
- Thymoglobulin	0 (0)
- Alemtuzumab	0 (0)
- Basiliximab	0 (0)
- Other	0 (0)
- No induction	0 (0)
Maintenance Immunosuppression	
- Tacrolimus	0 (0)

- Cyclosporine	0 (0)
- Mycophenolate	0 (0)
- Azathioprine	0 (0)
- mTOR inhibitors	0 (0)
- Steroid	0 (0)
