# Supplementary material for: Use of Machine Learning Consensus Clustering to Identify Distinct Subtypes of Kidney Transplant Recipients With DGF and Associated Outcomes
Source: Transpl Int. 2022 Dec 8;35:10810. doi: 10.3389/ti.2022.10810 (PMC9773391; doi:10.3389/ti.2022.10810)
Supplement: Supplementary file 2 [file DataSheet1.docx]

**Online Supplementary** - **Cluster Derivation**

We applied an unsupervised ML approach to develop clinical phenotypes of kidney transplant patients with DGF in the UNOS/OPTN database by conducting unsupervised consensus clustering.^1^ We performed consensus clustering analysis on the whole study population. We initially assessed the distribution and missingness in phenotyping variables. We included only variables that had <10% missing values. Subsequently, missing data were imputed through multiple imputation using multivariate imputation by chained equations (MICE), and Non-normal data were z-score normalized.^2^ Multiple imputation is a widely used approach to estimate variables when data are missing at random. By leveraging known patient characteristics and accounting for uncertainty in the multiple estimations of missing values, multiple imputation preserves sample size and reduces bias while examining association between variables.^3^

In the MICE procedure a series of regression models are run whereby each variable with missing data is modeled conditional upon the other variables in the data. This means that each variable can be modeled according to its distribution, with, for example, binary variables modeled using logistic regression and continuous variables modeled using linear regression. The algorithm is run multiple times in parallel to obtain m imputed datasets using MICE and values were averaged to obtain the final estimate.^2^ We subsequently applied clustering using the consensus cluster algorithm. The algorithm begins by subsampling a proportion of items and a proportion of features from a data matrix. Each subsample is then partitioned into up to groups (k) by a user-specified clustering algorithm. This process is repeated for a specified number of times. Pairwise consensus values, defined as ‘the proportion of clustering runs in which two items are grouped together’, are calculated and stored in a consensus matrix (CM) for each cluster. Clustering settings used were as follows: maximum number of clusters, 10; number of iterations, 100; subsampling fraction, 0.8; clustering algorithm, K-means; Euclidean distance).^1^ The number of potential clusters ranges from 2 to 10, to avoid producing an excessive number of clusters that would not be clinical useful. Pairwise consensus values, defined as ‘the proportion of clustering runs in which two items are [grouped] together, are calculated and stored in a CM for each k.^1^ Then for each k, a final agglomerative hierarchical consensus clustering using distance of 1−consensus values is completed and pruned to k groups, which are called consensus clusters.

The clustering algorithm is to maximize the potential number of clusters while maintaining high cluster consensus. The optimal number of clusters was determined by examining the CM heat map, cumulative distribution function, cluster-consensus plots with the within-cluster consensus scores, and the proportion of ambiguously clustered pairs (PAC).^4-5^ The within-cluster consensus score, ranging between 0 and 1, is defined as the average consensus value for all pairs of individuals belonging to the same cluster.^5^ A value closer to one indicates better cluster stability.^5^ PAC, ranging between 0 and 1, is calculated as the proportion of all sample pairs with consensus values falling within the predetermined boundaries.^4^ A value closer to zero indicates better cluster stability.^4^ We calculated the PAC using the strict criteria had the predetermined boundary of (0, 1), where a pair of individuals who had a consensus value greater than 0 or less than 1 was considered ambiguously clustered.^4^

Calculation of the standardized difference of each parameter used the cutoff of ±0.3 to show subgroup features with the key features for each cluster. All cluster derivation analyses were performed using R, version 4.0.3 (RStudio, Inc., Boston, MA; http://www.rstudio.com/), with the packages of ConsensusClusterPlus (version 1.46.0).^5^ We imputed missing data through multivariable imputation by chained equation (MICE) method.^2^ All analyses were two-tailed, and P value < .05 was considered statistically significant.

**References**

1. Monti S, Tamayo P, Mesirov J, Golub T. Consensus clustering: a resampling-based method for class discovery and visualization of gene expression microarray data. Machine learning. 2003: 91
2. Van Buuren S, Groothuis-Oudshoorn K. mice: Multivariate imputation by chained equations in R. Journal of statistical software. 2011: 1
3. Donders ART, Van Der Heijden GJ, Stijnen T, Moons KG. A gentle introduction to imputation of missing values. Journal of clinical epidemiology. 2006: 1087
4. Șenbabaoğlu Y, Michailidis G, Li JZ. Critical limitations of consensus clustering in class discovery. Sci Rep. 2014: 6207 [PMID: 25158761 10.1038/srep06207: 10.1038/srep06207]
5. Wilkerson MD, Hayes DN. ConsensusClusterPlus: a class discovery tool with confidence assessments and item tracking. Bioinformatics. 2010: 1572
